# Excessive Ingestion of Almond Milk Causes Severe Hypercalcemia and Acute Kidney Injury in a Patient With Chronic Kidney Disease

**DOI:** 10.31486/toj.24.0067

**Published:** 2025

**Authors:** Ahmad Bouhuwaish, Elgassi Ehnisch, Ahmed Abdullah Husayn Arhaym, Muner M. B. Mohamed, Juan Carlos Q. Velez

**Affiliations:** ^1^ University of Tobruk College of Human Medicine, Tobruk, Libya; ^2^ Department of Orthopedics, MKK Auguste-Viktoria-Klinik, Universitätsklinik für Allgemeine Orthopädie, Bad Oeynhausen, Germany; ^3^ Department of Internal Medicine, Tobruk Medical Center, Tobruk, Libya; ^4^ Department of Nephrology, Ochsner Clinic Foundation, New Orleans, LA; ^5^ The University of Queensland Medical School, Ochsner Clinical School, New Orleans, LA

**Keywords:** *Hypercalcemia*, *milk*, *milk substitutes*

## Abstract

**Background:**

Almond milk has a higher calcium content than cow's milk. Hypercalcemia after consuming almond milk has been reported in infants, but to our knowledge, we report the first case of almond milk–induced severe hypercalcemia in an adult.

**Case Report:**

A 66-year-old male with a history of diabetes and chronic kidney disease was referred to the emergency department because of laboratory results that showed severe hypercalcemia and acutely elevated serum creatinine. The family member who brought the patient to the hospital reported that he had displayed intermittent confusion. History revealed that 4 weeks prior, the patient had stopped his habit of consuming a gallon of cow's milk every day because of hyperglycemia. He switched to consuming a gallon of unsweetened almond milk every day, leading to severe hypercalcemia. Other causes of hypercalcemia were ruled out. Treatment with intravenous fluids and calcitonin normalized the patient's serum calcium level and improved his kidney function.

**Conclusion:**

The consumption of almond milk in large quantities is associated with the potential risk of hypercalcemia, especially in patients with chronic kidney disease. Careful consideration of the mineral content is recommended.

## INTRODUCTION

Hypercalcemia is a common condition but may have adverse consequences. Normal serum calcium levels are 8.7 to 10.5 mg/dL (2.0 to 2.5 mmol/L). Hypercalcemia is considered mild if the total serum calcium level is between 10.6 and 12.0 mg/dL (2.6 to 3.0 mmol/L) and severe if >14.0 mg/dL (>3.5 mmol/L). Primary hyperparathyroidism and neoplastic diseases are the principal etiologies of hypercalcemia.^1,2^

Milk-alkali syndrome is a form of hypercalcemia caused by the ingestion of calcium and absorbable alkali. In addition to hypercalcemia, metabolic alkalosis and renal insufficiency are also present in cases of milk-alkali syndrome.^3^ Hypercalcemia caused by excessive ingestion of almond milk is rare, and the reported cases are in infants.^4,5^ We present the unusual case of an adult patient whose excessive ingestion of almond milk resulted in severe hypercalcemia.

## CASE REPORT

A 66-year-old male was referred to the hospital by his primary care physician after routine laboratory testing showed hypercalcemia with serum calcium of 15.4 mg/dL and acute kidney injury with serum creatinine of 3.7 mg/dL. The family member who brought the patient to the hospital reported that the patient had been acting somewhat confused. The patient complained of stomach pain and constipation and showed signs of exhaustion and increased thirst. He had a medical history of diabetes mellitus type 2, essential hypertension, hypersensitivity lung disease, chronic kidney disease (CKD) stage 3b, peripheral arterial disease, and osteomyelitis of the toes status post amputation of the left second and fourth toes. His home medications included amlodipine, aspirin, atorvastatin, famotidine, insulin, losartan, and metformin.

According to the patient's family member, the patient had been consuming up to a gallon of cow's milk each day but stopped approximately 4 weeks prior because his blood sugar was not under control. The patient then began consuming a gallon of unsweetened almond milk (Almond Breeze [Blue Diamond Growers]) instead.

In the emergency room, the patient's rectal temperature was 36.9 °C, heart rate was 88 beats per minute, blood pressure was 150/86 mm Hg, respiratory rate was 18 breaths per minute, and oxygen saturation level on room air was 98%. The physical examination was unremarkable except for slurred speech and confusion.

The patient's laboratory workup (Table) was significant for severe hypercalcemia (serum calcium of 15.3 mg/dL) and an increase in serum creatinine to 3.4 mg/dL from baseline of 2.2 mg/dL. Vitamin D 1,25-dihydroxy was low at 10 pg/mL, and serum protein electrophoresis was negative. Renal ultrasound showed bilateral simple renal cysts without stones or hydronephrosis.

**Table.** Patient's Laboratory Data

		Testing Time Point				
Parameter	Reference Range	Baseline, 2/13/2023	Outpatient, 4/10/2023	Admission, 4/10/2023	Discharge, 4/13/2023	Follow-up, 5/2/2023
**Clinical Chemistry**						
Sodium, mmol/L	136-145	137	137	140	142	139
Potassium, mmol/L	3.5-5.1	5.0	4.4	3.8	3.4	4.9
Bicarbonate, mmol/L	23-29	23	21	23	19	21
Blood urea nitrogen, mg/dL	6-20	60	43	51	31	47
Creatinine, mg/dL	0.5-1.4	2.2	3.7	3.4	2.5	2.1
eGFR, mL/min/1.73 m^2^	>60	36	19	19	28	34
Calcium, mg/dL	8.7-10.5	9.8	15.4	15.3	9.5	10.0
Glucose, mg/dL	70-110	326	179	105	87	102
Phosphorus, mg/dL	2.7-4.5			3.1	3.1	
Alkaline phosphatase, U/L	55-135	125		94	82	92
Protein total, g/dL	6.0-8.4	7.1		7.1	5.9	7.2
Albumin, g/dL	3.5-5.2	3.4		3.8	3.2	3.8
Vitamin D 1,25-dihydroxy, pg/mL	20-79			10		
PTH, pg/mL	9.0-77.0			14		
Hemoglobin, g/dL	14.0-18.0	11.2		10.6	9.7	10.3
**Serum Electrophoresis**						
Kappa FLC, mg/dL	0.33-1.94			3.4		
Lambda FLC, mg/dL	0.57-2.63			1.77		
Kappa/lambda FLC ratio	0.26-1.65			1.92		
Immunofixation				No monoclonal peak		
Urine microalbumin/creatinine ratio	0-30			1,062.9		

eGFR, estimated glomerular filtration rate; FLC, free light chain; PTH, parathyroid hormone.

The patient was admitted to the hospital and started on intravenous fluid. He received 1 L of normal saline as a bolus, followed by infusion at a rate of 125 mL/h for 48 hours, and calcitonin injections of 336 U at a rate of 4 U/kg every 12 hours for 2 doses.

The patient's calcium level normalized after 2 days of treatment (serum calcium level of 9.5 mg/dL), and his serum creatinine level improved to 2.5 mg/dL. The patient was discharged home and advised to stop drinking almond milk. Arrangements were made for diabetes education and nutrition counseling. At 2-week follow-up after the patient had abstained from drinking almond milk, his primary physician reported that his symptoms were resolved, and the laboratory workup showed a serum calcium of 10.0 mg/dL and serum creatinine of 2.1 mg/dL.

## DISCUSSION

Severe hypercalcemia can present with vomiting, constipation, confusion, lethargy, and coma^6^ and is often complicated by acute kidney injury.^7^ Nephrocalcinosis, local renal vasoconstriction, and tubular dysfunction–induced water and sodium urinary losses can contribute to the development of acute kidney injury.^8^ Preexisting CKD could play a role in increasing an individual's susceptibility for hypercalcemia. In a cohort of 557 patients with CKD stage 4 and 5 who were not on dialysis and followed for 1 year, 13.4% of patients developed hypercalcemia.^9^ Thus, use of calcium supplements or consumption of calcium-containing food products may have greater potential to precipitate hypercalcemia among individuals with CKD vs in those who do not have CKD.

Plant-based milk alternatives are often advertised as being healthier and more natural than cow's milk. Almond milk is often fortified with calcium. Compared to cow's milk that contains 300 mg of calcium per 8 oz serving, Almond Breeze milk contains 450 mg of calcium per 8 oz serving.^10^ Our patient was consuming 1 gallon of cow's milk per day (4,880 mg of calcium), but because of hyperglycemia, he switched to 1 gallon of unsweetened Almond Breeze milk per day (7,232 mg of calcium).

In our patient, other causes of hypercalcemia were ruled out, including primary hyperparathyroidism, malignancies such as multiple myeloma, and vitamin D toxicity. Parathyroid hormone (PTH) was suppressed despite the patient's CKD, strongly supporting the notion that the acute hypercalcemic event was PTH-independent. No monoclonal peak was identified by serum protein electrophoresis, and the kappa/lambda light chain ratio was within the expected range for CKD. The vitamin D 1,25-dihydroxy level was not elevated. PTH-related peptide (PTH-rP) level was not obtained. However, the resolution of hypercalcemia after the cessation of almond milk consumption and the lack of recurrence constitute strong evidence against PTH-rP–induced hypercalcemia, Interestingly, metabolic alkalosis, a common feature in milk-alkali syndrome,^3,11^ was not present in this case.

Milk-alkali syndrome has become rare after widespread implementation of modern peptic ulcer disease therapies, but calcium-alkali syndrome has emerged as a result of the growing trends of osteoporosis therapy and broad availability of calcium-containing supplements. Health-seeking behavior may reintroduce patterns of food consumption that include milk products.^12^ Therefore, clinicians must be vigilant and properly educate their at-risk patients to avoid ingestion of a higher-than-recommended daily calcium load.

Severe hypercalcemia related to almond milk consumption is rare, and the reported cases are in pediatric patients.^4,5^ To our knowledge, ours is the first case of almond milk–related hypercalcemia to be reported in an adult.

## CONCLUSION

Our case demonstrates how consuming almond milk in large quantities can cause hypercalcemia and acute kidney injury. These conditions are dangerous, and proper history taking can lead to early detection and prompt intervention to achieve good patient outcomes. Systemwide education of physicians and other health care workers that almond milk has more calcium than cow's milk could have preventive benefits, particularly for patients with CKD.

## Notes

*This article meets the Accreditation Council for Graduate Medical Education and the American Board of Medical Specialties Maintenance of Certification competencies for Patient Care and Medical Knowledge.*
